# Strategies for mercury speciation with single and multi-element approaches by HPLC-ICP-MS

**DOI:** 10.3389/fchem.2022.1082956

**Published:** 2022-12-01

**Authors:** Laura Favilli, Agnese Giacomino, Mery Malandrino, Paolo Inaudi, Aleandro Diana, Ornella Abollino

**Affiliations:** ^1^ Department of Drug Science and Technology, University of Torino, Turin, Italy; ^2^ Department of Chemistry, University of Torino, Torino, Italy

**Keywords:** HPLC-ICP-MS, mercury, speciation, multi-element, ifenation, biological sample

## Abstract

Mercury (Hg) and its compounds are highly toxic for humans and ecosystems, and their chemical forms determine both their behavior and transportation as well as their potential toxicity for human beings. Determining the various species of an element is therefore more crucial than understanding its overall concentration in samples. For this reason, several studies focus on the development of new analytical techniques for the identification, characterization, and quantification of Hg compounds. Commercially available, hyphenated technology, such as HPLC-ICP-MS, supports the rapid growth of speciation analysis. This review aims to summarize and critically examine different approaches for the quantification of mercury species in different samples using HPLC-ICP-MS. The steps preceding the quantification of the analyte, namely sampling and pretreatment, will also be addressed. The scenarios evaluated comprehend single and multi-element speciation analysis to create a complete guide about mercury content quantification.

## 1 Introduction

As is well-known, mercury is regarded as a persistent and toxic element that impacts human and ecosystem health. The interaction between the metal and the environment starts when the Hg species are mobilized from natural deposits into the biosphere. Once it is mobilized, Hg can be transported through air, and water and, after undergoing various reactions, it can change its speciation, biomagnify, and bio-accumulate in food chains. Mercury species, especially organomercury compounds, can easily accumulate in fat tissues due to their lipophilic properties (Sundseth et al., 2017). Many studies show that mercury exposure may result in an important risk of cardiovascular disease, and it can have a negative impact on the reproductive and immune systems; in particular, the majority of Hg present in living organisms is in the form of methylmercury (CH_3_Hg^+^, abbreviated as MeHg hereafter), which is a developmental neurotoxin and has fetal neurotoxicity. The collection of literature data led to the development of greater awareness and the creation of new public health policies. The most common sources of mercury exposure are contaminated water and food, especially the tissues of animals at the top of the food chain subject to bio-accumulation phenomena. In addition, in some developing countries, where artisanal and small-scale gold mining (ASGM) is carried out, the inhabitants can inhale mercury vapors produced by the extraction process. High levels of mercury exposure are not related only to some populations but happen worldwide. However, the total mercury content in the environment compartments is low (Mergler et al., 2007; Gibb and O’Leary, 2014; Sundseth et al., 2017). Furthermore, element speciation is the key to comprehend an analyte’s potential toxicity, mobility, and bioavailability; this kind of study allows to obtain information not provided by total mercury quantification (Templeton and Fujishiro, 2017). For these reasons, it is essential to improve and develop new analytical strategies for determining different mercury species with high sensitivity and good accuracy. Most approaches rely on hyphenated techniques, coupling the separation of metal species by High-Performance Liquid Chromatography (HPLC) with their sensitive determination by Inductively Coupled Plasma Mass Spectrometry (ICP-MS).

The popularity of HPLC-ICP-MS is due to the ability to determine many elements in a single analytical run, the simple connection of separation and detection systems, and the versatility and sensitivity of the detector.

High-Performance Liquid Chromatogaphy is a separation technique that can quickly select non-volatile.

Compounds of high and low molecular weight. It can be used with different separation modes, providing high versatility; analytes can be divided depending on their chemical and physical properties such as polarity, solubility, ionic charge and size, and molecular mass. The most used approach is the reverse phase (RP), which involves the separation of molecules based on interactions between the analytes in the polar mobile phase and the lipophilic ligands attached to the stationary phase of the column. After the separation of the compound, the procedure requires a detector to correctly determine the concentration of each element species. ICP-MS is particularly suitable for this purpose, both for its intrinsic qualities and because it can be easily coupled with this chromatographic technique. It has low detection limits (<ppt levels), good linearity (up to 108 order of magnitude) and can examine one or more masses at the same time with high resolution. The advantage of using these two techniques in a sequence is to combine an effective separation of the species under examination with a sensitive and versatile detector (Ponce de León et al., 2002; Gao et al., 2012).

This review addresses the quantification of mercury species in several matrices by HPLC-ICP-MS. Since the sensitivity and accuracy of the response depend on the pretreatment procedures, these steps will also be discussed. Both speciation and multielement analysis will be considered. Papers published between 2000 and 2022 were reviewed. A bibliographic search on Web of Science was carried out using mainly the keywords “HPLC-ICP-MS”, “Mercury speciation” and “Multielement speciation” In literature, there are lots of procedures to investigate single element speciation or total concentration. However, a single element approach requires a huge investment of time and capital. Switching to a multielement approach permits to decrease the volume and the amount of reagent required and reduce the analysis time. In some cases, it is even possible to develop multielement speciation analysis, optimizing the use of time and resources. However, building a procedure to analyze different elements is challenging, because the pretreatment, determination, and separation conditions must be optimized for all analytes at once (Wolf et al., 2011; Sun et al., 2015). Thanks to this review it will be possible to select the best working conditions in the pretreatment, separation, and quantification phases according to the purpose of the different methodologies.

## 2 Sample pretreatment

The sample pretreatment is a crucial issue in trace Hg speciation. The procedures for total Hg determination or Hg speciation analysis are easily affected by interconversion of species, contamination, and analyte losses, especially if they involve steps of transport and storage of the samples. The affinity of inorganic Hg species to surface adsorption and their high volatility increases the probability of analyte loss. Sources of contamination can be identified in storage containers and stabilizing reagents used to decrease Hg evaporation or wall adsorption. It is also necessary to pay attention to the possible inter-conversion reactions between Hg species, which are due to the procedures used and the matrix type. For example, part of Hg(II) may transform into MeHg through extraction, derivatization or measurement processes (Castillo et al., 2010; Abad et al., 2017). Different reviews, such as the one of Brown at all (Pandey et al., 2011), paid particular attention to the necessity for clean sampling procedures, and proper sample storage.

### 2.1 Water

Pyrex and Teflon (PTFE or FEP) are the best materials for containers in both storage and processing phases for water samples, because of their low affinity with metal species. There are several efficient methods for cleaning sampling vials and other equipment that comes into contact with samples, to avoid contamination (e.g., aqua regia, chromic acid, nitric acid, and BrCl) (Bravo et al., 2018). Water samples are filtered with 0.22 or 0.45 μm pore size filters to eliminate particulate materials (Amde et al., 2016) and pretreated to stabilize volatile compounds, as Hg° and dimethylmercury (DMHg), and reduce their loss. If the purpose of the procedure is to determine total mercury, it is sufficient to acidify the sample with HCl or HNO_3_ or stabilize it with the addition of an oxidant (BrCl) to preserve the analytes and to prevent the formation of microbes; whereas, for MeHg, the samples can be acidified with HCl or stored unpreserved deep-frozen. Changing the initial conditions of the solution favors interconversion reactions: therefore, to perform a speciation analysis, acids or oxidants cannot be used for stabilization. Instead of stabilizing liquid samples for transport and storage, it is possible to selectively adsorb the analyte on a solid phase (e.g., gold, Carbotrap, Tenax, etc.). These processes can be advantageously performed in the field. *In situ* preconcentration procedures are a valid alternative to overcome the problems that occur during sample transport and storage and lower the limits of detection (LODs) (Leermakers et al., 2005).

### 2.2 Air

There are several ways to accumulate gaseous or particulate-bound mercury, among these, each researcher chooses the most accurate, precise, and suitable method for the type of analysis of interest. Usually, a huge volume of air is required to obtain a detectable quantity of Hg, since the concentrations of Hg are extremely low in this matrix. Therefore, it is useful to adopt pre-concentration to get concentrations above the LOD. For these reasons, the first step of sampling gaseous Hg or Hg in particulate matter is usually to draw a measured volume of air through a collection material able to retain the species of interest (see below). The process should be performed with pumps designed for trace-level pollutant sampling. The volume of air sampled (in L) must be measured to calculate the concentration in L o m^3^ of air, for example with a dry test meter (DTM) placed in the vacuum line, between the pump and the sample box. Most of the studies focus on the determination of total gaseous mercury (TGM or Hg°), reactive gaseous mercury (RGM), organic species (such as MeHg and DMHg), or mercury bound to particulate matter (Hgp). These compounds can be collected separately using different materials as reported in the literature (Lu and Schroeder, 1999; Pandey and Kim, 2008); for example, MeHg and DMHg are retained by Carbotrap (Graphitized carbon) or Tenax TA (Polymer of 2,6-diphenylphenol), indeed Hg° can be trapped with gold amalgamation (Leermakers et al., 2005; Pandey et al., 2011).

### 2.3 Biota and sediment samples

The majority of Hg present in living organisms is in the form of MeHg, which is easily absorbed from the gastrointestinal tract and has a high biomagnification factor (up to 106) in the food chain because of its high liposolubility (Gao et al., 2012). The samples of biota tissue are usually stored at low temperatures, sometimes lyophilized, or sterilized. It is advisable to avoid repeated freezing and unfreezing cycles to avoid MeHg decomposition (Leermakers et al., 2005). Sediment samples are commonly packed in PE bags and transported to the laboratory in a cooler at low temperatures. In the second step, they are usually dried, homogenized, sieved, and stored at 4°C in high-density PE containers cleaned with acid (Amde et al., 2016). Soils can be processed and analyzed fresh or after a lyophilization or drying procedure. The presence and the influence that these pretreatments have on the determination of MeHg, and other analytes are subjects of debate in the scientific community. Some studies do not find differences between fresh sediments and dried (lyophilized) sediments (Muhaya et al., 1998), while others have found lower concentrations in the wet samples than in the dried ones (Muhaya et al., 1998; Leermakers et al., 2005). Further investigation in this field is required. Sediments and biota samples are solid; therefore, the analyte must be extracted from these matrices with adequate recovery. In literature, a wide variety of combinations of strong oxidizing acids, and elevated temperatures and pressure are reported and suggested for the determination of the total Hg content. The main issues of this phase are sample contamination, volatilization, and adsorption losses, especially during elevated temperature and pressure digestion procedures (Collasiol et al., 2004). The majority of the recommended methods rely on microwave-assisted digestion using closed vessels with acid and/or oxidant solvent (in this case concentrated sulphuric and nitric acid, and 30% hydrogen peroxide) (Murphy et al., 1996). This approach is characterized by rapid sample preparation and reduced risk of contamination from the laboratory environment and analyte loss, but the relatively high amount of reagent used can cause an increase of the blank values and the detection limits. There are various alternatives such as ultrasound-assisted leaching. The collapse of gas or vapor bubbles creates areas of high temperature (which increase solubility and diffusivity) and pressure (which promote penetration and transport) at the interface between an aqueous or organic phase and a solid matrix. This condition, combined with the oxidative energy of radicals created during sonolysis of the solvent (hydroxyl and hydrogen peroxide for water), results in high extractive power with a low reagent volume (Ruiz-Jiménez et al., 2003; Collasiol et al., 2004). At present, there has been considerable research focused on reagents for extracting mercury species. These works aim to select the most appropriate reagent based on the nature of the investigated samples and to validate the analyses performed (Issaro et al., 2009).

## 3 Speciation analysis

The research on the elemental speciation of an analyte is crucial to understanding its toxicity, mobility, and bioavailability in the environment (Templeton and Fujishiro, 2017). Organic Hg compounds, especially MeHg, are considered the most toxic forms of mercury since they are lipophilic and, for this reason, easily bioaccumulated and biomagnified in the food chain. However, all the mercury emitted into the environment can go through biogeochemical transformation processes and be converted into MeHg. For these reasons MeHg is the subject of numerous studies that deal with different matrices and ecosystems. Other organomercury species, such as dimethylmercury (CH3-Hg-CH3, abbreviated as DMHg hereafter), are only considered by a few studies, due to their different chemical-physical properties and consequent lower impacts on ecosystems. In fact, DMHg is a neutral, volatile organomercurial present in marine environments, that is not expected to bioaccumulate to levels of concern. (Baya et al., 2015; West et al., 2022) Consequently, the different mercury species have different impacts on the health of the biota and the ecosystem. Therefore, monitoring total mercury content in the environment does not provide enough information, and speciation analysis is needed to evaluate mercury’s toxicity and health risks. These methods are usually based on hyphenated techniques between chromatographic separation and atomic spectrometric detection as HPLC-ICP-MS (Gao et al., 2012; Cheng et al., 2018).

### 3.1 Preconcentrating procedures

Highly sensitive detection of potentially toxic elements has always been pursued, due to their low concentration range in environmental and biological samples. Consequently, it is important to study simple, efficient, and economic ways to increase the sensitivity of mercury determination. In the last few years, several pre-concentration processes have been proposed; they provide an improvement in sensitivity and can also separate the analytes from the sample matrix to decrease interferences. In Table 1, different procedures are listed: among these, those based on solid phase extraction (SPE) are the most tested ones. It has different benefits such as elevated recovery and enrichment factors, easy management and automation, low consumption of organic solvents, and high flexibility, which allows studying the application of numerous stationary phases with new eluents. For example, in 2019, Jia et al. used Zwitterion-functionalized polymer microspheres (ZPMs) as the sorbent phase of SPE, finding high enrichment factors and low limits of detection for Hg(II), MeHg, and ethyl-mercury (EtHg) (Table 1).

**TABLE 1:** Preconcentration approaches adopted for the determination of low concentrations of mercury species.

Separation method	Matrix	Stationary phase	Solvent	Sample volume (ml)	Analytes	LOD (ng/L)	Enrichment factor	Linear range (ng/L)	Separation time (min)	References
SPE	Freshwater	GO@SiO_2_	5 ml of 10 mM benzoic acid	10	Hg(II)	0.005 ± 5%	1963	0.05–50	5	Yang et al. (2019)
					MeHg	0.006 ± 5%	1881			
					EtHg	0.009 ± 5%	1794			
SPE	Tuna fish and lobster hepatopancreas	multiwalled carbon nanotubes functionalized with poly-l-methionine (polymet-MWCNTs)	1 ml of 10% v/v HCl +0.1% v/v ME	20	Hg(II)	0.5 ± 5%	>190	0.5–100	10	Labutin et al. (2016)
					MeHg					
SPE	Environmental waters	N-ZPMs	60 mM ammonium acetate and 0.15% l-cysteine (L-Cys) solution	40	Hg(II)	0.78 ± 4.1%	105	5–500	5	Jia et al. (2019)
					MeHg	0.63 ± 4.1%	98			
					EtHg	0.49 ± 4.1%	98			
MSPE	Fish protein	Fe_3_O_4_@COF-S-SH	5% HCl solution containing 1‰ (m/v) thiourea	200	Hg(II)	0.96 ± 5.4%	370	3.20–1,000	10	Bi et al. (2021)
					MeHg	0.17 ± 3.2%	395	0.55–1,000		
					EtHg	0.47 ± 4.3%	365	1.57–1,000		
SPE	Seawater	Thiol-functionalized silica microspheres (SH–SiO_2_)	60 ml of 100 mM l-cysteine (Cys) + 0.1 M HCl	5	Hg(II)	0.019 ± 4%	830	0.2–200	8.5	Cheng et al. (2015)
					MeHg	0.013 ± 4%	916			
					EtHg	0.014 < 4%	883			
CEC	Seawater	—	l-cysteine solution	30	Hg(II)	0.042 ± 5.1%	1,250	0.5–100	<3.5	Jia et al. (2012)
					MeHg	0.016 ± 4.4%				
					EtHg	0.008 ± 3.6%				
CPEL	Environmental waters	Directly-thiolated graphene (G-SH)	3molL−1 HCl	40	Hg(II)	3.8 ± 5.2%	78	13–500	—	Li et al. (2017)
					MeHg	1.3 ± 4.7%	77	4.5–500		
MSPE	Fish samples, environmental, waste and tap water	Fe_3_O_4_@SiO_2_@γ-mercaptopropyltrimethoxysilane (γ-MPTS)	0.1 mol L−1 HNO3 containing 4.0% (m/v) thiourea	100	Hg(II)	0.74 ± 2.7%	179	5–5,000	8	Zhu et al. (2017)
					MeHg	0.67 ± 4.0%	179			
					PhHg	0.49 ± 9.0%	174			
MSPE	Water sample	D@RGO- Fe_3_O_4_ adsorbing dithizone on the surface of the magnetite-reduced graphene oxide composites	0.5 mol L−1 l-cysteine, 0.1 mol L−1 HCl, and 10% CH3OH	400	Hg(II)	0.48 ± 4.2%	400	1.6–500	40	Li et al. (2019)
					MeHg	0.17 ± 3.8%	380	0.6–500		
MSPE	Water sample, soil, rice and fish	Fe_3_O_4_@SiO_2_@GMA-S-SH	0.5 ml 0.1 mol L−1 nitric acid and 4% thiocarbamide	200	Hg(II)	0.40 ± 7.4%	367	1.6–5,000	7	He et al. (2019)
					MeHg	0.49 ± 8.1%	380			
					PhHg	1.4 ± 8.3%	329	5.0–5,000		
HF-LLLME	Atmospheric particles, lake water, fish, plant and sediment	toluene containing 5 g/L PAN as extraction phase	5 mmol/L Na2S2O3 aqueous solution as the acceptor phase	5	Hg(II)	4.1 ± 6.4%	236	0.02–5	30	Chen et al. (2015)
					MeHg	2.9 ± 5.6%	233	0.01–5		
					EtHg	4.7 ± 8.7%	221	0.02–5		
					PhHg	5.6 ± 10.6%	229			

CEC, cation-exchange column; CPEL, Cloud point extraction-like method; MSPE, magnetic solid-phase extraction; HF-LLLME, fiber-liquid–liquid–liquid microextraction.

### 3.2 HPLC separation conditions

There are different types of approaches in liquid chromatography used for different applications depending on the characteristic of the analytes, such as relative polarity, solubility, and molecular mass. Such approaches include normal and reversed-phase, reversed-phase ion-pair, micellar, ion-exchange, size exclusion, and chiral LC. However, when LC is hyphenated to ICP-MS, some of them create problems in terms of plasma stability, waste disposal, or safety. For example, using a non-polar mobile phase is challenging HPLC-ICP-MS, so reversed-phase LC is now the most used method of partition chromatography. Usually, the stationary phases are made of siloxanes bound to some hydrophobic substituent groups that contain eighteen, eight, or one carbon atom(s): with this separation technique, the analytes are separated according to their different hydrophobicity, adjusting the selectivity of the separation by regulating the types and proportions of the components of the mobile phase. Binary, tertiary, or quaternary combinations of solvents may be used to achieve the desired selectivity (Sutton and Caruso, 1999). Other typologies of liquid chromatography can provide some difficulties in combination with ICP-MS as a detector, such as size exclusion chromatography, due to the high salt concentration that is often used in the mobile phase (Beauchemin, 2020). Table 2 summarizes the chromatographic conditions adopted for the separation of Hg species. Several authors suggest the use of reverse phases with l-cysteine and mercaptoethanol, methanol, and ammonium acetate in various combinations as mobile phases. l-cysteine and 2-mercaptoethanol are commonly used as thiol ligands for Hg in a mixture because the retention time was too long when 2-mercaptoethanol was used alone, and it is not possible to separate completely the mercury species with l-cysteine only. Ammonium acetate is often used as a buffer to control the pH, and the use of methanol leads to an increase in Hg detection sensitivity and a decrease in the retention time for concentrations up to 4–5% v/v. According to several authors, the reason for this effect is that methanol at low concentrations provides appropriate elution strength for the 2-mercaptoethanol or cysteine complexes of mercury species because these complexes are quite hydrophobic, while higher concentrations cause a loss sensitivity due to a reduction in plasma energy (Chen et al., 2009a; de Souza et al., 2010; Rodrigues et al., 2010).

**TABLE 2:** Chromatographic conditions adopted for the separation of mercury species.

Matrix	Stationary phase	Mobile phase	Analytes	LOD (µg/L)	Mobil phase flow rate (ml min−1)	Sample loop (µL)	Separation time (min)	References
Urine	RP: PerkinElmer Brownlee Analytical C18 column (4.6 × 150 mm, 3 µm)	5% MeOH, 0.4% l-cysteine and 0.05% 2-mercaptoethanol	MeHg	0.06	1	60	10	Tsoi, et al. (2010)
			EtHg					
Urine	CEC: polyhydroxy methacrylate gel (Shodex, MSpak SP-80 4B, 4.6, 50 mm, 8 µm)	250 mM NaCl and 100 mM GSH (pH 3.0)	Hg(II)	0.1	0.5	50	10	Chen, et al. (2009a)
			MeHg	0.03				
Blood Plasma	RP: Brownlee Columns C8 (4.6 × 33 mm, 3 µm)	MeOH: (0.5% 2-mercaptoethanolþ 0.05% HCO_2_H), (3:97)	Hg(II)	0.0012	1.2	100	8	de Souza, et al. (2013)
			MeHg	0.004				
			EtHg	0.005				
Blood Plasma	RP: Shim-Pack XRODS C18 column (2.0 × 30 mm, 2.2 μm) (Shimadzu Europa GmbH, Duisburg, Germany)	MP A: 0.1% HCO_2_H	Hg(II)	-	0.3	10	9	Trümpler et al. (2009)
		MP B: MeOH	MeHg	-				
Blood	RP: C18 (4, 150 mm, 5 µm) and a precolumn RP18 (3.2 15 mm, 7 µm) Brownlee Columns PerkinElmer, United States)	0.4% l-cysteine, 0.06 M NH_2_Ac, 0.05% 2-mercaptoethanol and 5% MeOH	Hg(II)	0.25	1	100	<5	Rodrigues et al. (2010)
			MeHg	0.1				
Hair	RP: C18 (4 × 150 mm, 5 µm) and a pre-column RP18 (3.2 × 15 mm, 7 µm)	0.4% l-cysteine, 0.06 M NH_2_Ac, 0.05% mercaptoethanol and 5% MeOH	Hg(II)	16	1.4	100	<9	de Souza et al. (2010)
			MeHg	66				
			EtHg	94				
Hair	RP: Symmetry Shield RP18 column, 3.9, 150 mm, 5 μm, Waters, Milford, United States)	5% MeOH, 0.1% 2-mercaptoethanol and 0.06 M NH_2_Ac	Hg(II)	0.2 ± 3.0%	1	50	13	Wang et al. (2007)
			MeHg	0.2 ± 5.8%				
Hair	RP: Novapak C18 (3.9, 150 mm, 4 µm) (Waters HPLC Columns, Milford, MA, United States)	5% MeOH, 0.1% 2-mercaptoethanol w/0.06 M NH_2_Ac (pH 6.8)	Hg(II)	0.005	1	20	11	Morton, et al. (2002)
			MeHg					
Water and Hair	RP: Supelco (United States) Discovery C18 (4.6, 15 mm, 5 µm)	MeOH: H2O with 0.1 mM DDTC (90:10)	Hg(II)	0.004	0.7	20	<6	Chen et al. (2009a)
			MeHg	0.01				
Water, Hair, and fish	RP: Discovery C18 column (4.6, 15 mm, 5 µm) (Supelco, PA, United States)	MeOH: CH_3_CN: H_2_O with 0.1 mM DDTC (35: 40:25)	Hg(II)	0.006	0.8	20	14	Chen et al. (2009c)
			MeHg	0.008				
			PhHg	0.013				
Environmental waters	RP: microbore column, Interchrom ODB (100 mm × 1 mm, 3 µm)	Gradient elution using methanol and l-cysteine at pH 3.0	Hg(II)	11	0.07	20	13	Castillo et al. (2006)
			MeHg	23				
			EtHg	8				
			PhHg	32				
Natural waters	RP: 100 × 2.1 mm Alltima HP C-18 3 µm particle size	0.5% l-cysteine (m/v) and 0.05% 2-mercaptoethanol (v/v)	Hg(II)	0.07	0.2	0.1–5	4	Chang et al. (2007)
			MeHg	0.02				
Sea water	RP: C18 column (150 × 3.2 mm id) packed with Spherisorb S5 ODs2 (5 μm, Phenomenex, Macclesfield, Cheshire, United Kingdom)	1%v/v acetonitrile and 0.005% 2-mercaptoethanol in 0.06 moll-' ammonium acetate	HgCl2	0.25	1	100	13	Bloxham et al. (1996)
			MeHgCl	0.25				
			EtHgCl	0.75				
Seafood	RP: C-18, Phenomenex Synergi HydroRP, 150 mm × 4.6 mm	0.1% w/v l-cysteine + 0.1% w/v l-cysteine·HCl·H_2_O	Hg(II)	7 ± 5%	2	50	6	Hight and Cheng, (2006)
			MeHg	5 ± 9%				
Biological samples	CEC: PRP-X200 polymer-based cationexchange column	50 mmol l1 pyridine, 0.5% w/w l-cysteine and 5% v/w MeOH, at pH 2	Hg(II)	0.05	1	20	8	Vallant et al. (2007)
			MeHg	0.08				
Sediment and biological tissues	RP: ODS RP C18 Hypersil 80 mm × 4.6 mm	acetonitrile/water 65%/35%, by volume, adjusted to pH 5.5 by ammonia acetate	Hg(II)	2	1	50	10	Falter and Ilgen, (1997)
			MeHg	3				

RP, reverse phase; CEC, cation-exchange column.

### 3.3 ICP-MS conditions

Hyphenated techniques have the disadvantage that the compatibility of the procedure with all involved techniques must be considered. For instance, as reported above, the eluent composition can strongly affect ICP-MS efficiency. Usually, the mobile phases chosen for mercury speciation analysis comprise buffers, organic modifiers, ion pairs, or chelating agents. Organic solvents, like methanol, can cause carbon deposition on the sampling and skimming cones or plasma discharge instability. These problems can be toned down by optimizing the working conditions; in the study of (Chen et al., 2009b) the authors decided to increase plasma forward power, cool the spray chamber, and add optional gas flow containing oxygen to mitigate the methanol effect. They obtained a rise in the signal-to-noise ratio for mercury, with a maximum value at the optional gas flow rate of 0.3 L min−1. In addition, even after running a mobile phase (35% methanol and 40% acetonitrile) for 8 h in the HPLC-ICP-MS system, carbon deposition on the sampling cone could hardly be observed (Chen et al., 2009c).

The choice of the mobile phase can be affected by the pre-concentration technique applied before the chromatographic step. For example, using cloud point extraction (CPE), water-soluble mercury species are converted into water-insoluble chelates through a suitable chelating reagent: to separate these hydrophobic chelates, reverse-phase o HPLC with high organic solvent content must be adopted. Also in this case, (Chen et al., 2009d) (Chen et al., 2009b) optimized working conditions. The plasma forward power was set at 1500 W to stabilize plasma discharge and the temperature was maintained at -5°C through a Peltier cooling system: this approach permitted to remove most of the organic solvent from the sample aerosol. As in the previous example, they applied an optional gas flow containing oxygen to lower the carbon deposition on the sampling cone: the signal-to-noise ratio of mercury reached the maximum at the optional gas flow rate of 0.3 L min−1 (Chen et al., 2009b).

## 4 Multi-element speciation analysis

Although most of the procedures, allow for speciation analysis of a single element, this approach is not practical. Developing a separate method for each element and the need to carry out a large number of analyses to determine the speciation of several analytes in a sample requires a large investment of time and capital (Sun et al., 2015). Instead, multi-element speciation procedures decrease drastically the analysis time, the volume of reagent needed, and, therefore, the amount of chemical waste. The multi-element approach seems to be a suitable tool, especially in evaluating human exposure to different toxic elements species simultaneously in polluted regions and examining elements that coexist or interact with each other (Wolf et al., 2011). Several methodologies allowing for multi-elemental speciation analysis have been developed (Marcinkowska and Barałkiewicz, 2016a).

However, elaborating methods for multi-element speciation analysis in a single run is challenging since it is necessary to simultaneously optimize the pretreatment separation and detection conditions for all analytes. It is necessary to adjust preconcentration and HPLC/ICP-MS conditions considering the behavior of the various species to gain: 1) retention of each analyte on a chromatographic column and its elution in a reasonable time; 2) a complete separation of analytical signals; 3) the maintenance of the stability of the different species throughout the whole analytical procedure; 4) elimination of potential interferences; 5) sufficiently low detection limits (Sun et al., 2015).

### 4.1 Preconcentration procedures

As pointed out single-element speciation analysis, directly evaluating the element content in environmental samples is often challenging due to the complexity of the matrices and/or the low concentration range: for instance, the concentration range for total mercury in unpolluted natural water could be 0.03–90 ng/L, while the lead one is also in the order of magnitude of several ng/L (Song et al., 2021). In these cases, it is useful to apply pre-concentration procedures to increase the concentration level. In the literature, there are some examples of methods that could be used for this purpose; they are the same as those mentioned for single-element preconcentration: for example, cloud point extraction (Jia et al., 2019), ionic liquid extraction (Falish Ramandi and Shemirani, 2015), liquid-liquid micro-extraction (Akramipour et al., 2018) and SPE/SPME (Płotka-Wasylka et al., 2016). Among these sample pretreatment procedures, SPE is one of the most adequate to readily couple with HPLC-ICP-MS as discussed above (Moreno et al., 2010).

Lots of new procedures and materials have recently been tested, as shown in Table 3. Song et al., in 2022 built a method to simultaneously determine Cr, Cd, Hg, and Pb species at ng/l level by integrating online SPE into HPLC-ICP-MS. They retained the elements on a C_18_ column and eluted by the mobile phase subsequently used for HPLC separation. The procedure reached low LOD values (0.001–0.007 ng L), satisfactory enrichment factors (827–2,656 folds), and good repeatability (<4.0%). These values ensured good applicability of the method to the simultaneous measurements of trace Cr, Cd, Hg, and Pb compounds in water (Song et al., 2022).

**TABLE 3:** Preconcentration approaches adopted for the determination of low concentrations of various element species.

Method	Matrix	Stationary phase	Solvent	Sample volume (ml)	Analytes	LOD (ng/L)	Enrichment factor	Linear range (ng/L)	Separation time (min)	References
SPE	water (tap, mineral, pond, river and purified)	C18 column	5 mM Cys (pH 2.0) in aqueous solution	10	Cd(II)	0.003 ± 2.3%	1,608	0.1–100	8	Song et al. (2022)
					Cr (III)	0.006 ± 2.5%	1,074			
					Cr(VI)	0.006 ± 2.5%	1,074			
					EtHg	0.001 ± 3%	2,656			
					Hg(II)	0.003 ± 4%	1701			
					MeHg	0.001 ± 3.8%	2,220			
					Pb(II)	0.004 ± 3.9%	1,261			
					triethyl lead (TEL)	0.004 ± 3.2%	1,362			
					trimethylead (TML)	0.007 ± 2.6%	827			
SPE	water (tap, rain, mineral, pond, river and purified)	1 cm C18 enrichment column preconditioned with 10 ml of 1 mM 2-mercaptoethanol at 10 ml min1	5 mM l-cysteine (Cys) at pH 2.5	10	EtHg	0.002 ± 2.2%	1866	0.1–100	13.2	Song et al. (2021)
					Hg(II)	0.001 ± 2.8%	2,485			
					MeHg	0.002 ± 1.6%	1984			
					Pb(II)	0.011 ± 2.4%	459			
					triethyl lead (TEL)	0.004 ± 3.9%	1,627			
					trimethylead (TML)	0.003 ± 4.5%	1,248			
SPE	water (spring, lake, and tap)	Carbon nanotube sponges (CNT sponges) functionalize with Sodium diethyldithiocarbamate (DDTC)	acetonitrile	200	Co.(II)	0.089 ± 1.49%	200	0.5–100	5	Wang et al. (2016)
					Cu(II)	0.061 ± 3.60%				
					Hg(II)	0.690 ± 1.27%		3–400		
HF-LPME	water (tap, river and estuarine) and human blood plasma	Q3/2 polypropylene (Accurel Q3/2, Membrana, Wuppertal, Germany)	-	10	MeHg	230 < 15%	27	-	20	Moreno et al. (2013)

*SPE, solid-phase extraction;* HF-LPME*,* hollow fiber membrane extraction*.*

### 4.2 HPLC separation conditions

One of the main tasks to obtain an efficient procedure of multi-element speciation analysis is the selection of the separation parameters. The optimization of the chromatography phase is challenging because conditions for different elements species may vary significantly (Marcinkowska, et al., 2015). The key is to choose the best stationary and mobile phase, starting from the data in the literature and applying the necessary changes to adapt the procedures to each case study. Zhang et al. (2020) selected the best mobile phase for the separation of As, Hg, and Pb. They report that lots of studies use alkyl ammonium bases/salts such as tetrabutylammonium hydroxide (TBAH) and hexadecyl trimethyl ammonium bromide as mobile phases in an ion-pairing RPC mechanism for the separation of arsenic species. For the speciation of mercury instead, the mobile phase used often contains thiol compounds (Cys and 2-mercaptoethanol, etc.), while negatively charged alkyl sulfonic acids/salts (sodium 1-pentanesulfonate SPS, sodium dodecyl sulfate, etc.) are commonly adopted for the separation of cationic lead forms. They then investigated the behavior of each possible mobile phase for each single element speciation. in the next phase of the investigation, they utilized mobile phases containing two-chemical mixed solutions of the three compounds (TBAH, Cys, and SPS). The best results were obtained with a mixture of TBAH and Cys, which is suitable for the simultaneous separation of all species of the three analytes (Chang et al., 2007; Marcinkowska and Barałkiewicz, 2016b). Other studies, comprising different analytes and methodologies, are shown in Tables 4, 5.

**TABLE 4:** HPLC conditions for the determination of low concentrations of various element species.

Matrix	Stationary phase	Mobile phase	Analytes	LOD (µg/L)	Flow rate (ml/min)	Sample loop (µL)	Separation time (min)	References
Water (tap, river and estuarine) and human blood plasma	Phenomenex Luna C18 (250 mm × 4.60 mm, 5 m, 100 ° A pore size) (California, United States)	Gradient of methanol (mobile phase B) and 0.14% (v/v) 2-mercaptoethanol in 50 mM ammonium acetate at pH 4.6 (mobile phase A)	MeHg Hg(II) Se(II)	230 < 15% 110 < 15% 131 < 15%	-	10	8	Moreno et al. (2013)
Water (tap, mineral, pond, river and purified)	15 cm AQ C18 column (5 μm × 4.6 mm ID, Welch Materials) preconditioned by sulfur-containing compounds	5 mM Cys (pH 2.0) in aqueous solution	Cd(II)	0.003 ± 2.3%	1	10	8	Song et al. (2022)
			Cr (III)	0.006 ± 2.5%				
			Cr(VI)	0.006 ± 2.5%				
			EtHg	0.001 ± 3%				
			Hg(II)	0.003 ± 4%				
			MeHg	0.001 ± 3.8%				
			Pb(II)	0.004 ± 3.9%				
			triethyl lead (TEL)	0.004 ± 3.2%				
			trimethylead (TML)	0.007 ± 2.6%				
water (tap, rain, mineral, pond, river and purified)	25 cm AQ C18 column (5 mm 4.6 mm i.d., Welch Materials (Shanghai) Inc.)	5 mM l-cysteine (Cys) at pH 2.5 in the 0e1 and 4e15 min and 5 mM Cys þ 0.5 mM tetrabutyl ammonium hydroxide solution at pH 2.5 in the 1e4 min	EtHg	0.002 ± 2.2%	-	10	13.2	Song et al. (2021)
			Hg(II)	0.001 ± 2.8%				
			MeHg	0.002 ± 1.6%				
			Pb(II)	0.011 ± 2.4%				
			triethyl lead (TEL)	0.004 ± 3.9%				
			trimethylead (TML)	0.003 ± 4.5%				
water (spring, lake, and tap)	Unitary C18 100A column (4.6 mm × 250 mm, 5 μm) from Acchrom (Beijing, China)	acetonitrile and water (80:20, v/v)	Co.(II)	0.089 ± 1.49%	1	50	12	Wang et al. (2016)
			Cu(II)	0.061 ± 3.60%				
			Hg(II)	0.690 ± 1.27%				
Urine and serum	two columns: RP = Phenomenex Luna C18 column, 250 mm × 4.60 mm, 5µm, 100 x A pore size. Chiral = Astec Chirobiotic T column, 250 mm 4.6 mm, 5 µm	A = 0.075% tetraethylammonium chloride, pH 4.5. Mobile phase B = 0.1% 2-mercaptoethanol, 0.06 M ammonium acetate, 5% methanol	D-SeMet	1 ± 6%	1	100	27	Yin et al. (2013)
			L-SeMet	1 ± 9%				
			Se-MeSeCys	2 ± 4%				
			Se(IV)	1 ± 6%				
			Se(VI)	1 ± 6%				
			MeHg+	1 ± 6%				
			Hg2+	0.3 ± 6%				

**TABLE 5:** Selected applications of HPLC-ICP-MS to the determination of multiple species in different matrices.

Elements	Sample	Chromatographic separation	HPLC operating conditions	ICP-MS detection system	Interferences Figures of merits ref	References
	1. Analytes—inorganic/organic compounds 2. Matrix	1. Column	1. Mobile phase flow rate (ml min1)	1. Type of detection system	1. LD values (mgL1)	
		2. Elution type	2. Column temperature (°C)	2. Monitored isotopes	2. Precision as a RSD of peak area (%)	
		3. Mobile phase	3. Injection volume (ml)		3. Recoveries (%)	
			4. Retention time (min)			
			5. Total analysis time (min)			
Pb, Hg	1. Et3Pb+—triethyl lead (II) chloride, Ph3Pb+– triphenyl lead (II) chloride, MeHg+—methylmercury (II) chloride, EtHg+—ethylmercury (II) chloride, PhHg+—phenylmercury (II) chloride	1. Waters Symmetry C8 microbore column (50 mm 1.0mm, 3.5 µm)	1. 0.1	1. Elan 6,000, PerkinElmer/SCIEX with direct injection high efficiency nebulizer (DIHEN)	1. No data	Rodushk and ödman, (2001)
			2. No data	2. 200Hg, 202Hg, 207 Pb, 208 Pb	2. No data	
			3. 10			
	2. Standard solutions	2. Isocratic	4. 1.49 for MeHg+, 1.88 for EtHg+, 4.19 for PhHg+, 1.75 for Et3Pb+, 7.79 for Ph3Pb+			
		3. 7.5 mM sodium pentanesulfonate and sodium dodecanesulfonate in 20% acetonitrile, pH ¼ 3 with 5% HNO3	5. 10			
Pb, Hg	1. PbII—lead (II) nitrate, trimethyl-Pb+– trimethyl lead (II) chloride, triethyl-Pb—triethyl lead (II) chloride, HgII—mercury (II) nitrate, methylHg—methylmercury (II) chloride, ethyl-Hg—ethylmercury (II) chloride	1. Alltech microbore column C18, (150 mm 1 mm, 5 µm)	1. 0.12	1. Perkin-Elmer SCIEX ELAN 6100 DRCII with high efficiency nebulizer (DIHEN, 170AA)	1. No data	Acon, et al. (2001)
	2. Fish samples	2. Isocratic	2. Room temperature	2. 200Hg, 202Hg, 206 Pb, 207 Pb, 208 Pb	2. No data	
		3. 0.2% (v/v) 2-mercaptoethanol, 174.2 mg L1 1-pentanesulfonate, 12% (v/v) MeOH and 1 mg L1 EDTA, pH 2.8	3. 5			
			4. 0.67 for PbII, 3.17 for trimethyl-Pb, 14.83 for triethyl-Pb, 8.08 for HgII, 5.75 for methyl-Hg and 15.08 for ethyl-Hg			
			5. 16.7			
Pb, Hg	1. Et3Pb+–triethyl lead (II) chloride, Me3Pbþ+– trimethyl lead (II) chloride, MeHg+—methylmercury (II) chloride, EtHg+—ethylmercury (II) chloride, PhHg + phenylmercury (II) chloride	1. CETAC Technologies RP C18 (500 mm 1.6 mm)	1. 0.1	1. Sciex Elan Model 250 with Direct Injection Nebulizer (DIN)	1. No data	Chang, et al. (2007)
	2. Standard solutions	2. Isocratic	2. No data	2. 202Hg, 208 Pb	2. No data	
		3. 5 mM ammonium pentanesulfonate in 20:80 v/v ACN-H2O (pH ¼ 3.4)	3. 2			
			4. 2.0 for Et3Pb+, 0.9 for Me3Pb+, 1.2 for MeHg+, 1.8 for EtHg+, 4.4 for PhHg+			
			5. 6.0			
	1. Analytes—inorganic/organic compounds	1. Column	1. Mobile phase flow rate (ml min1)	1. Type of detection system	1. LD values (mgL1)	
	2. Matrix	2. Elution type	2. Column temperature (°C)	2. Monitored isotopes	2. Precision as a RSD of peak area (%)	
		3. Mobile phase	3. Injection volume (ml)		3. Recoveries (%)	
			4. Retention time (min)			
			5. Total analysis time (min)			
Se, Hg	1. TMeSeI—trimethyl selenonium iodide, (SeIICys)2—selenocystine, SeIIUr—selenourea, SeIIMet—selenomethionine, MeHg+—methylmercury (II) chloride, Hg2+ mercury chloride	1. 1. RP18 column (3.9 mm 150 mm, 5 µm)	1. No data	1. Thermo Elemental X7 ICP-MS	1. 1 40Ar40Ar þ interfering with 80Se +	Li et al. (2007)
		2. Gradient	3. 100		2. Collision cell technique (CCT) with 92.72% He and 7.29% H2 as a collision gas	
	2. Urine samples	3. A: 0.1% heptafluorobutanoic acid (HFBA), 0.3% methanol, B: 3% v/v methanol, 0.06 M ammonium acetate, 0.1 v/v 2-mercaptoethanol	4. 2.0 for TMeSeI, 2.5 for (SeIICys)2, 4.5 for SeIIUr, 8.0 for SeIIMet, 20.0 for MeHg+, 22.5 for Hg2 +	2. 78Se, 80Se, 202Hg		
			5. 27.0			
Se, Hg	1. 1. SeIV—sodium selenite, SeVI—potassium selenate, D-SeIIMet—Dselenomethionine, L-SeIIMet—Lselenomethionine, MeSeIICys methylselenocysteine, MeHg + methylmercury (II) chloride, Hg2+—mercury chloride	1. Phenomenex Luna C18 column (250 mm 4.60 mm, 5 μm, 100 Å pore size) and Astec Chirobiotic T column (250 mm 4.6 mm, 5 µm) e connected using a column-switching system	1. No data	1. HP 4500	1. No data	Moreno et al. (2010)
	2. Urine and serum samples	2. Gradient	2. No data 3.100	2. 77Se, 78Se, 202Hg	2. No data	
		3. A: 0.075% tetraetylammonium chloride, pH 4.5; B: 0.1% 2-mercaptoethanol, 0.06 M ammonium acetate, 5% methanol	3. 3.82 for Se-MeSeIICys, 4. 35 for SeIV, 8.00 for SeVI, 12.68 for LSeIIMet, 13.00 for DSeIIMet, 23.82 for MeHg+, 25.55 for Hg2+			
			4. 27			

### 4.3 ICP-MS condition in the multi-element speciation analysis

#### 4.3.1 Reaction/collision cells

Although several articles had previously been published concerning multi-element analyzes of metals and metalloids by ICP-MS, from 2000 onwards the number of studies increased thanks to the introduction of the Dynamic Reaction Cell (DRC) to the market. This has proved to be an effective tool for eliminating spectral interferences, paving the way for the use of quadrupole ICP-MS for multi-elementary analyzes. High-resolution ICP-MS were already capable to reach this performance, but their high cost and their scarce robustness prevented their widespread diffusion. As already specified, in multi-elemental studies by HPLC-ICP-MS, only one set of operating conditions must be chosen for all analytes simultaneously. This is a complex task because each element has its interfering ions, with different mechanisms of elimination. Furthermore, the effect of this process could impact even elements not suffering from interferences, and it must be considered during the optimization of the DRC. All details concerning collision/reaction cells working conditions applied in multi-elemental speciation analysis are presented in Table 5 (Marcinkowska and Barałkiewicz, 2016a).

#### 4.3.2 Other approaches to dealing with interferences

Since the ICP-MS detector can detect more isotopes for an analyte, it is possible to quantify the elements by analyzing the isotopes least burdened by interferences with polyatomic ions. However, the use of less abundant isotopes leads to a decrease in instrumental sensitivity with a consequent increase in the detection limit. For example, the chromium isotopes most abundant in nature are ^52^Cr with 83.8% and ^53^Cr with an abundance of 9.5% (Markiewicz et al., 2015). In the study performed by Mulugeta et al., the less abundant isotope of Cr was used to overcome the interferences deriving from the ^40^Ar^12^C^+^ connection; the corresponding LoD was relatively high but sufficient to enable the quantification of the element in the samples under examination (leachates from cement-based material) (Mulugeta et al., 2010). Alternatively, it is possible to correct the spectral interferences with mathematical methods, especially suitable for monatomic elements such as Ar, for which it is not possible to analyze a minority isotope. Optimization of chromatographic separation can also help; for example, (Roig-Navarro et al., 2001) noticed that in their work argon chloride did not give interference problems on arsenic species determination because arsenic and chloride were separated chromatographically (Roig-Navarro et al., 2001).

## 5 Competitive alternatives to HPLCICP-M

Gas chromatography (GC) coupled to mass spectrometry (GC-MS) or to inductively coupled plasma mass spectrometry (GC–ICP-MS) appear to be hyphenated techniques capable of competing with the performance of HPLC-ICP-MS. GC is another high resolving chromatographic separation technique capable of effectively separating volatile and semi-volatile compounds. Several methods have been developed for the accurate quantification of organomercury species through GC; many of them include a derivatization step to convert these non-volatile compounds into volatile species suitable for analysis by GC with sensitive detectors. (Zachariadis et al., 2008) developed a method to for the simultaneous determination of MeHg and inorganic Hg in human body fluids using GC-MS. Analytes were derivatized by *in situ* ethylation with sodium tetraethylborate (NaBEt_4_) in aqueous solutions and extracted with a headspace solid phase microextraction (HS-SPME) procedure. MeHg and inorganic Hg species extracted by spiked human urine, saliva, and serum were separated by GC and detected by MS, obtaining good repeatability and LOQ. (Zachariadis et al., 2008; Lusilao-Makiese et al., 2012) instead, tested a method to investigate the distribution of Hg in coal from South Africa, using a four-step sequential leaching procedure and isotope dilution with GC-ICP MS. Although the process is quite complex, it permits to determine inorganic Hg and MeHg efficiently; chromatograms also showed unknown Hg peaks which were identified as EtHg. (Lusilao-Makiese et al., 2012) Simultaneous isotope dilution analysis with GC-MS and GC-ICP-MS on certified bivalve samples, were compared by Cavalheiro et al. (2014). Overall, both techniques performed well in measuring inorganic Hg and MeHg concentrations in the CRM, showing excellent linearity and precision. Using GC ICP-MS it is possible to obtain better sensitivity**,** making it possible to work with high solvent volumes (low pre-concentration factors). However, it must be considered that GC-MS is less sophisticated than GC-ICP-MS, requiring less qualified personnel to operate the equipment and lower cost of maintenance. (Cavalheiro et al., 2014) Isotope dilution GC-MS and HPLC-ICP-MS were compared by (Wang et al., 2013) for the determination of MeHg in fish samples. The authors did not find any differences between the performances of the two techniques, nevertheless, they have highlighted how HPLC-ICP-MS is the most used due to its high sensitivity, no need for prior derivatization, i.e. possibility of separating native Hg species. In addition, isotope dilution GC-MS is more time-consuming, since it requires overnight digestion and signal-matched isotope dilution spiking. (Wang et al., 2013)

## 6 Conclusion

This review presents an overview of recent analytical strategies for determining different mercury species with high sensitivity and good accuracy with HPLC-ICP-MS. It must be pointed out that the reliability of the results depends also on the pretreatment procedures. Through these years, numerous preconcentration methods were developed for mercury species and multielement speciation analysis. Among these options, SPE is the simplest preconcentration system to connect with a liquid chromatographic instrument, and the most tested one in literature because of its several advantages: high recoveries and enrichment factors, ease of management and automation, use of low amounts of organic solvents, and high flexibility, which allows its application on a wide range of research contexts. Its flexibility also helps in choosing the HPLC phases, which is one of the most critical tasks to achieve an efficient procedure.

l-cysteine and 2-mercaptoethanol are commonly used in the mobile phase for mercury speciation analysis, as thiol ligands for Hg, in a mixture with ammonium acetate, as a buffer, and methanol, to increase Hg detection sensitivity and reduce the retention time. Detector conditions are consequently optimized to minimize any problems caused by the mobile phase mixture’s components (such as organic solvents). On the other hand, the conditions used in multielement speciation analysis methods depend on the target elements. The optimal pretreatment and separation conditions may vary significantly depending on the element in exam: for this reason, the method must be adjusted to obtain compromise conditions suitable for all the species involved. In literature, there are numerous examples with different analytes and applications from which to start to optimize a proper method for specific studies.

In conclusion, it is possible to affirm that the numerous studies on methodologies and the frequent improvement of the available instruments allow to obtain fast and effective analysis procedures for mercury speciation. Furthermore, because of ICP-MS’s multi-element capabilities, it is possible to extend the single-element speciation procedures to determine the species of multiple elements in a single analytical run, reducing the time and costs needed.
